# Supplementary material for: Rats with Long-Term Cholestasis Have a Decreased Cytosolic but Maintained Mitochondrial Hepatic CoA Pool
Source: Int J Mol Sci. 2023 Feb 22;24(5):4365. doi: 10.3390/ijms24054365 (PMC10001988; doi:10.3390/ijms24054365)
Supplement: Supplementary file 1 [file ijms-24-04365-s001.zip › ijms-2206874-supplementary.pdf]

Table S1. Fractionation of the liver homogenate. Rats were bile duct ligated for 4 weeks (BDL rats, n = 9) or sham-operated control rats (n = 5). After euthanasia by cervical dislocation, the liver was removed and homogenized. After homogenization, the liver was fractionated by serial centrifugation as described in Methods. Enzyme activities were determined by spectrophotometric methods and protein was measured according to Lowry with bovine serum albumin as standard. Data are presented as the mean±SEM. * $p < 0.05$ vs. control.

	Control (n=5)	BDL rats (n=9)
**Liver homogenate**		
Protein (mg/g liver)	190±9	215±12
Citrate synthase (µmol/min/g liver)	12.2±0.9	10.8±0.5
Lactate dehydrogenase (µmol/min/g liver)	315±22	335±19
**Liver mitochondria**		
Mitochondrial protein isolated	26.1±1.8	22.1±1.7*
Citrate synthase (nmol/min/mg mitochondrial protein)	163±10	161±13
Citrate synthase isolated (µmol/min/g liver)	4.24±0.23	3.54±0.29*
Citrate synthase recovery (%)	34.7±1.6	32.8±1.9
Total mitochondrial protein (mg/g liver)	74.9±7.7	67.1±5.6
Lactate dehydrogenase (µmol/min/mg mitochondrial protein)	0.021±0.002	0.024±0.002
Contamination with cytosolic protein (mg/mg mitochondrial protein)	0.007±0.001	0.008±0.002
**Liver cytoplasm**		
Cytosolic protein isolated	89.3±5.7	93.5±6.0
Lactate dehydrogenase (µmol/min/mg cytosolic protein)	3.00±0.20	3.15±0.14
Lactate dehydrogenase isolated (µmol/min/g liver)	268±30	295±16
Lactate dehydrogenase recovery (%)	85±3	88±4
Total cytosolic protein (mg/g liver)	105±11	106±8
Citrate synthase (nmol/min/mg protein)	1.62±0.20	2.42±0.20*
Contamination with mitochondrial protein (mg/mg cytosolic protein)	0.010±0.002	0.016±0.002
